# Ultra-Short Duration Hypothermia Prevents Intracranial Pressure Elevation Following Ischaemic Stroke in Rats

**DOI:** 10.3389/fneur.2021.684353

**Published:** 2021-09-20

**Authors:** Daniel Omileke, Debbie Pepperall, Steven W. Bothwell, Nikolce Mackovski, Sara Azarpeykan, Daniel J. Beard, Kirsten Coupland, Adjanie Patabendige, Neil J. Spratt

**Affiliations:** ^1^The School of Biomedical Sciences and Pharmacy, University of Newcastle, Callaghan, NSW, Australia; ^2^Hunter Medical Research Institute, New Lambton, NSW, Australia; ^3^Department of Neurology, John Hunter Hospital, Hunter New England Local Health District, New Lambton, NSW, Australia

**Keywords:** intracranial pressure, hypothermia, middle cerebral artery occlusion, clinical translation, animal model

## Abstract

There is a transient increase in intracranial pressure (ICP) 18–24 h after ischaemic stroke in rats, which is prevented by short-duration hypothermia using rapid cooling methods. Clinical trials of long-duration hypothermia have been limited by feasibility and associated complications, which may be avoided by short-duration cooling. Animal studies have cooled faster than is achievable in patients. We aimed to determine whether gradual cooling at a rate of 2°C/h to 33°C or 1°C/h to 34.5°C, with a 30 min duration at target temperatures, prevented ICP elevation and reduced infarct volume in rats. Transient middle cerebral artery occlusion was performed, followed by gradual cooling to target temperature. Hypothermia to 33°C prevented significant ICP elevation (hypothermia ΔICP = 1.56 ± 2.26 mmHg vs normothermia ΔICP = 8.93 ± 4.82 mmHg; *p* = 0.02) and reduced infarct volume (hypothermia = 46.4 ± 12.3 mm^3^ vs normothermia = 85.0 ± 17.5 mm^3^; *p* = 0.01). Hypothermia to 34.5°C did not significantly prevent ICP elevation or reduce infarct volume. We showed that gradual cooling to 33°C, at cooling rates achievable in patients, had the same ICP preventative effect as traditional rapid cooling methods. This suggests that this paradigm could be translated to prevent delayed ICP rise in stroke patients.

## Introduction

Stroke is the second leading cause of death worldwide and the number one cause of permanent disability in adults (1, 2). A good collateral flow network is associated with better neurological outcome after stroke (3, 4). The collateral circulation provides residual perfusion to the “at-risk” penumbral tissue and slows down the progression of the infarct core.

Dramatic elevations in intracranial pressure (ICP) occur after experimental ischaemic stroke in animals - in young and aged rats, as well as rats of different strains (5–7). Our preliminary data indicates a significant ICP rise also occurs in stroke patients at 24 h (8). This ICP rise is a potential mechanism for collateral failure associated with delayed infarct expansion, worsening stroke outcome in patients (9, 10). We have previously shown that an increase in ICP causes a dramatic decrease in collateral blood flow and may therefore be responsible for collateral failure (9). Experimental studies have shown that ICP elevation occurs even after small strokes (5, 6, 11, 12) which may explain why it has gone unnoticed in patients with minor stroke, as ICP is normally only measured in large hemispheric stroke, due to the invasive nature of the procedure (13). Short-duration therapeutic hypothermia is a potent, easily implemented strategy that we have recently shown to have robust efficacy in preventing ICP elevation 24 h after stroke in rats (5–7). Multiple previous studies have shown that hypothermia also reduces infarct volume and improves functional outcome after experimental stroke (14), although at the time of these studies the effect on ICP was unknown, and the presumed mechanism of neuroprotection was by modification of a wide range of cell death mechanisms (14, 15). The effects of hypothermia on ICP elevation therefore suggests that direct effects on tissue perfusion via collateral vessels may be an important mechanism of hypothermic cytoprotection. Several early-phase clinical stroke trials using hypothermia as a treatment measure have shown feasibility. However, there is a significant mismatch in cooling duration used in clinical studies, from that shown to be effective in many experimental studies (16). Clinical trials of hypothermia have an average cooling duration of 24 h, with many maintaining hypothermia for up to 72 h (15). In contrast, the vast majority of experimental studies have cooled for 1–6 h (14). Additionally, rodent studies typically achieve target temperature within 10–20 min (14), a rate that cannot be achieved when cooling a human. The necessity of long-duration hypothermia to achieve therapeutic outcome in stroke is questionable. Current protocols resulted from very early clinical studies in patients with extremely large, “malignant” middle cerebral artery (MCA) infarcts, in whom rebound ICP elevation during rewarming was extremely problematic, and on occasion fatal (17). However, these durations are logistically extremely challenging, and increase the risk of complications such as pneumonia (18). Moreover, our experimental data suggests short-duration cooling may prevent rebound ICP elevation, thus obviating the need for very prolonged rewarming. The mismatch between protocols shown to be effective in experimental studies, and those tested in clinical trials, needs to be addressed if there is any hope of translating therapeutic hypothermia for stroke treatment.

We hypothesized that a clinically achievable gradual cooling protocol may require even less time at target temperature than current methods to prevent ICP elevation post-stroke. A milder target temperature for a short duration is easier to achieve and would potentially increase feasibility. We previously showed that hypothermia to 35°C did not lead to a significant increase in ICP 24 h after stroke. However, a slight ICP rise was seen, suggesting that 35°C may be close to the threshold for hypothermia ICP rise prevention (6).

We aimed to determine the benefits of ultra-short duration hypothermia at target temperature on both ICP elevation and infarct volume reduction post-stroke. For this, two hypothermia target temperatures (33 and 34.5°C) were investigated to identify the most feasible hypothermia regimen that still effectively prevented ICP elevation. The “ultra-short” duration refers to the 30 min at target temperatures, which is far shorter than previous studies of short duration cooling (14). We chose 34.5°C, slightly lower than the mildest effective target of 35°C from our previous work, to allow for our use of a gradual paradigm with less time at target temperature than in the previous study. ICP was measured epidurally using a fiber-optic catheter system. Epidural measurements were preferred in this study due to the increased risk of brain damage associated with other methods of ICP monitoring (19). Any damage to the brain has the potential to alter ICP, and therefore influence the primary outcome of this study. Moreover, previous studies have found that epidural ICP recordings correlate well with intraventricular recordings (20, 21), which are the “gold standard” of ICP measurements in humans (19). Additionally, the use of a fiber-optic probe provides high fidelity ICP signals when inserted and sealed in the epidural space (19, 22).

## Materials and Methods

### Animals and Experimental Protocol

Adult male (11–12 weeks old) outbred Wistar rats (*n* = 23, Animal Services Unit, University of Newcastle) weighing 280–320 g were used for this study. Animals were housed under standard conditions in a 12 h light-dark cycle with unlimited access to food and water. All experimental procedures were in accordance with the Australian Code of Practice for the Care and Use of Animals for Scientific Purposes and were approved by the Animal Care and Ethics Committee of the University of Newcastle (A-2013-343). This study was reported in accordance with the ARRIVE guidelines (23).

Rats were initially anesthetised in 5% isoflurane in 50:50 N_2_:O_2_ in an induction chamber. Anesthesia was maintained with 2–2.5% isoflurane in the same gas mix and delivered via a custom, low dead space face mask with cross flow of gases. Incision sites were shaved, cleaned and injected subcutaneously (s.c.) with 2 mg/kg 0.05% Bupivacaine (Pfizer, Sydney, Australia). Body temperature was regulated throughout the surgery with a rectal temperature thermocouple (RET-2, Physitemp Instruments Inc, Clifton, New Jersey, USA). The femoral artery was cannulated with a catheter consisting of 1 and 2 French silicone tubing for continuous monitoring of arterial blood pressure and heart rate and for measurement of arterial blood gases (i-STAT, Abbot, New Jersey, USA). Animals were randomized by sealed envelope to hypothermia treatment (33 or 34.5°C), or normothermia. After hypothermia treatment/ normothermia, rectal paracetamol (250 mg/kg, GlaxoSmithKline, Brentford, UK) was administered for recovery and overnight pain relief. Animals were also injected with saline (2 × 1.5 mL, s.c.) to prevent dehydration and were returned to their cages with free access to softened laboratory chow and water.

### Implantation of Datalogger Device

A 2 cm longitudinal incision was made along the right abdominal region, proximal to the right thigh. The incision was made deep enough to expose the space at the ventral thigh crease. Haemostats and forceps were used to create a pocket under the skin that was large enough to hold the device. The temperature monitoring datalogger (Maxim, San Jose, USA) was inserted into the pocket and secured by closing the muscle and skin with 5-0 silk sutures. Core body temperature was chosen as the desired temperature measurement location because it is minimally invasive and allows for post-operative and overnight monitoring. Additionally, we have previously compared core, rectal and brain temperature in rats and found that these temperatures track together during hypothermia induction, maintenance and rewarming (unpublished observations).

### Intracranial Pressure and Laser Doppler Measurement

**Figure 1:**
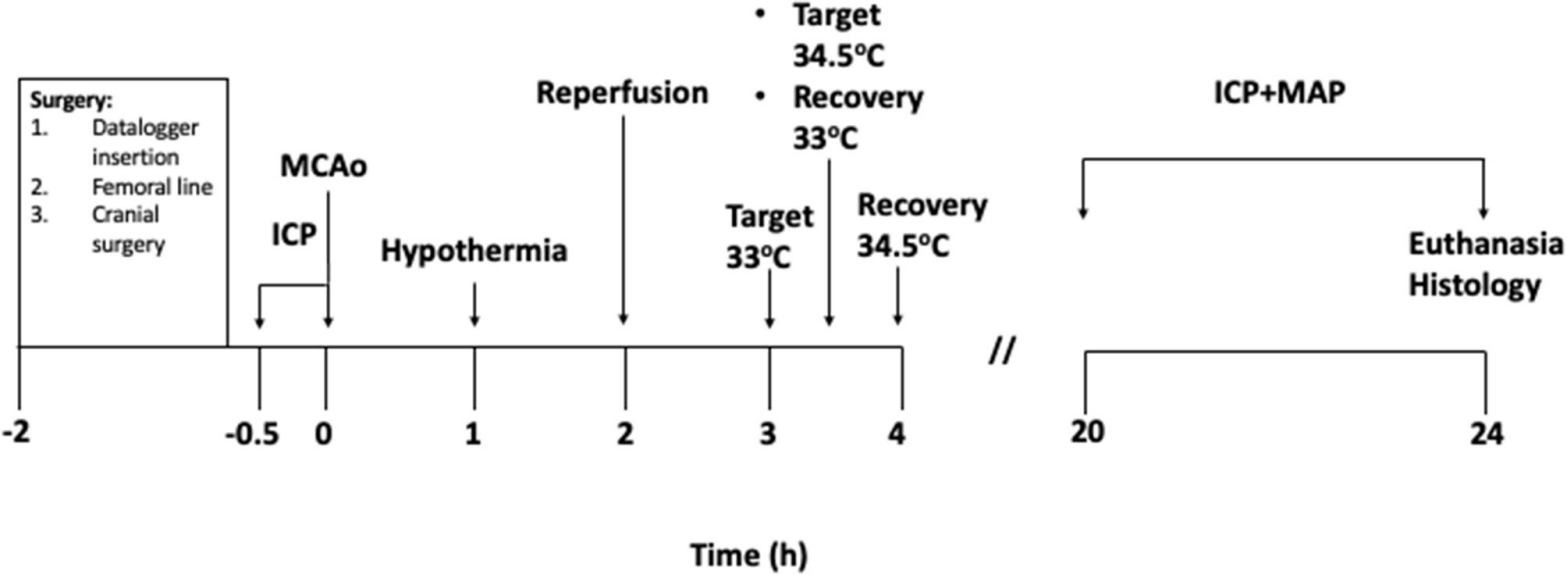
Experimental protocol (in hours). Baseline intracranial pressure (ICP) recordings were taken for 30 min prior to starting MCAo. 0 h signifies the moment of vessel occlusion. Hypothermia treatment was initiated 1 h after occlusion in the hypothermia groups. Target temperature was achieved 2 h after hypothermia initiation in the 33°C group and maintained for 30 min. Target temperature was achieved 2.5 h after hypothermia initiation in the 34.5°C group and maintained for 30 min. Animals in both groups were rewarmed to 35°C immediately after the maintenance period and recovered from surgical procedures. Normothermia animals were maintained at 37°C.

Cranial surgery was performed according to previously described methods (22). To summarize, the ICP probe (OpSens Fiber Optic Pressure Sensors, Canada) was inserted epidurally into a saline filled, polyether ether ketone (PEEK) screw (Bregma 2 mm posterior and 2 mm lateral) in the left parietal bone. Tissue perfusion in the territory supplied by the right middle cerebral artery was monitored during middle cerebral artery occlusion (MCAo) and reperfusion using laser Doppler flowmetry (LDF). The LDF probe (Moor Instruments, UK) was inserted into a second hollow PEEK screw (Bregma 2 mm posterior and 5 mm lateral) in the right parietal bone. For ICP and LDF recordings, the screws were secured with dental cement and an airtight seal was created around each probe using a caulking material (Silagum, Gunz Dental, Germany). Correct placement of the ICP probe was confirmed by a response to abdominal compression which was observed on both ICP and arterial blood pressure waveforms. ICP was monitored at pre-stroke baseline and again at 20–24 h post-stroke (Figure 1). Cerebral perfusion pressure (CPP) is the difference between mean arterial pressure and ICP, and was therefore calculated by subtracting these two values. To account for minor variations between the baseline ICP and CPP of the 3 experimental groups, change in ICP from baseline to 24 h (ΔICP), and change in CPP from baseline to 24 h (ΔCPP) were used for all ICP and CPP analyses.

### Middle Cerebral Artery Occlusion

Transient middle cerebral artery occlusion (MCAo) was carried out according to our established protocol (24, 25). To summarize, a 6 cm length of monofilament nylon suture (3 mm length × 0.38 mm O.D silicone) was inserted into the ligated right external carotid artery. The filament was advanced 20 mm through the internal carotid artery, avoiding the pterygopalatine artery, until resistance was felt, and a drop in perfusion units (>50% drop from baseline) on the LDF was observed which indicated that the middle cerebral artery has been occluded. At 2 h post-occlusion, reperfusion was achieved by retracting the monofilament through the internal carotid artery approximately 18 mm until the silicone tip was visible in the external carotid artery stump.

### Hypothermia Treatment

**Figure 2:**
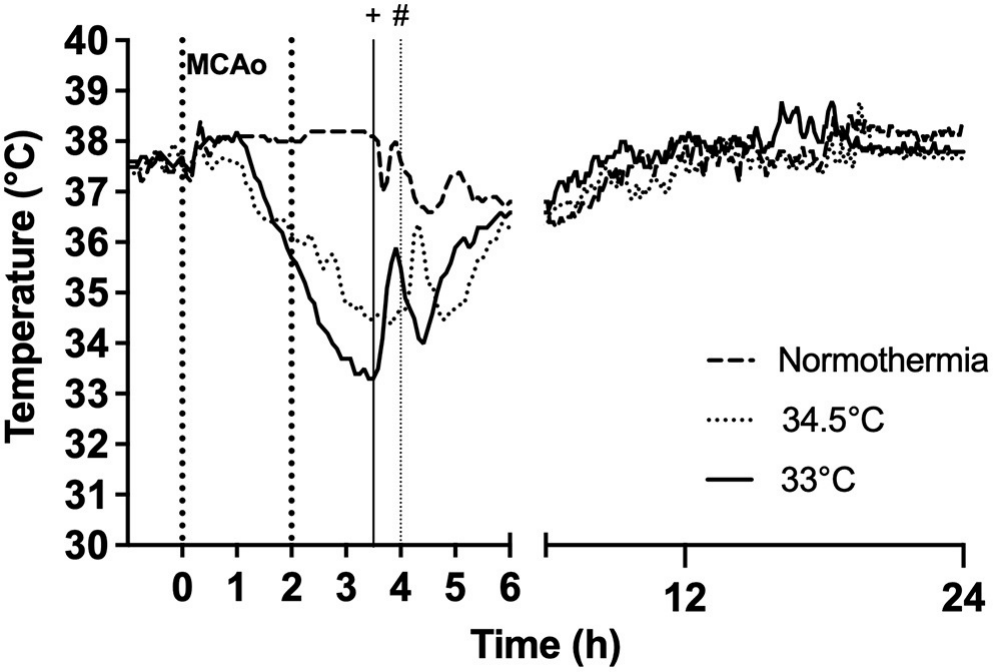
Effect of gradual cooling protocol on core body temperature (datalogger) in rats subjected to hypothermia at 33°C and 34.5°C. Vertical dotted lines at 0 h to 2 h refer to duration of occlusion. Hypothermia was initiated at 1 h post-occlusion in both 33°C and 34.5°C groups. Target temperature was reached at 3 h post-occlusion in the 33°C group and 3.5 h post-occlusion in the 34.5°C group. “+” indicates rewarming was initiated at 3.5 h in the 33°C group and “#” indicates rewarming initiation at 4 h in the 34.5°C group.

After 1 h of MCAo, hypothermia-treated animals were cooled gradually by reducing the temperature of the heat mat in small increments (26) to a target core temperature of 33 or 34.5°C. Animals were cooled to target over 2 h in the 33°C group (rate 2°C/h) and over 2.5 h in the 34.5°C group (rate 1°C/h), and maintained at target for 30 min. This gave an overall cooling duration of 2.5 h and 3 h, respectively. No external cooling was necessary as anesthesia prevents normal regulation of core body temperature (Figure 2). For recovery and rewarming, core body temperature was increased to 35°C by adjusting the heat mat. Animals were placed in a cage half over a warming pad (Passwell, South Australia) to allow for thermoregulation back to normothermia. Animals in the 33°C took 127 ± 36.4 min to thermoregulate back to normothermia. Animals in the 34.5°C group took 116 ± 39.4 min to thermoregulate back to normothermia. Animals in the normothermia group were maintained at 37°C for the duration of the surgery.

### Histological Analysis and Infarct Volume Measurement

Animals were euthanised 24 h post-stroke onset. They were transcardially perfused with saline and their brains were removed and sectioned into 6 coronal slices using a rat brain matrix, each of 2 mm thickness.

Triphenyltetrazolium chloride (TTC) (Sigma-Aldrich, Missouri, USA) staining was performed to confirm the presence of ischaemic stroke by identification of infarcted tissue. The slices from each brain were incubated for 12 min at 37°C in 2% TTC. TTC was used for early confirmation of infarct, however, haematoxylin and eosin (H&E) staining was then used on the same tissue for infarct volume quantification. Infarct volume quantification was carried out using our routine procedure (27). Tissues were fixed, processed, paraffin embedded and cut at 10 μm coronal sections. Images were scanned using a high-resolution scanner (Aperio, Vista, CA, USA) and analyzed by an investigator blinded to treatment allocation. Infarct (corrected for oedema) was calculated (Aperio ImageScope) by subtracting the measured interhemispheric volume difference from the measured infarct volume for each side. Infarct volumes were corrected for oedema by applying the formula: corrected infarct volume (mm^3^) = infarct volume × (contralateral volume/ipsilateral volume). Oedema was calculated by infarct volume minus corrected infarct volume (6).

### Exclusion Criteria and Statistical Analysis

Subarachnoid hemorrhage (SAH), equipment malfunction and lack of >50% LDF drop at occlusion were pre-specified exclusion criteria.

A sample size calculation was performed using pilot and previous data (5, 6) (G^*^Power version 3.1) which indicated that 4 animals per group were required to detect a 10 mmHg difference in ΔICP between the hypothermia and normothermia groups (ΔICP = mean peak ICP – mean baseline ICP) with standard settings of alpha 0.05, power 0.8. A sample size of 5 animals per group was decided upon to allow for any outlier effects. Statistical analyses were performed using GraphPad Prism version 8.2.1. Data were tested for normal distribution using the Shapiro-Wilk normality test. One-way analyses of variance (ANOVA) were used to compare differences between the treatment groups (main effect) followed by Tukey's *post-hoc* test for multiple comparisons between groups when a significant difference was found. Student's *t*-test (paired) were performed to compare changes from baseline. Statistical significance was accepted at *p* < 0.05. Data are presented as mean ± SD unless otherwise stated.

## Results

A total of 15 animals were included in this study: 5 treated with hypothermia to 33°C, 5 treated with hypothermia to 34.5°C and 5 normothermia controls. A total of 8 animals were excluded. Reasons for exclusion were lack of sufficient LDF drop (*n* = 5), SAH detected post-mortem (*n* = 1) and unexpected death from surgical complications (*n* = 2).

### Gradual Cooling Slowly Reduces Body Temperature

Both cooling protocols achieved a gradual decrease of core body temperature to target (Figure 2). Core temperatures during the 30 min at target were 33.2 ± 0.27°C and 34.5 ± 0.22°C in the two hypothermia groups. Normothermia animals were maintained at normal core body temperature for the duration of this period. In the hypothermia groups, animals reached target at 117.8 ± 26.9 min in the 33°C group and 152.2 ± 2.3 min in the 34.5°C group. Physiological parameters are presented in Table 1.

**Table 1:** Physiological parameters.

	**Baseline**			**24 h**		
	***Normothermia***	***33°C***	***34.5°C***	***Normothermia***	***33°C***	***34.5°C***
BP (mmHg)	101.9 ± 8.0	103.0 ± 7.2	103.8 ± 4.8	103.0 ± 7.8	112.8 ± 12.1	98.7 ± 7.4
RR (BPM)	66.9 ± 5.4	63.2 ± 4.8	64.4 ± 4.8	63.0 ± 7.3	60.0 ± 5.1	67.6 ± 12.8
HR (BPM)	421.8 ± 27.6	433.7 ± 22.6	448.1 ± 12.1	386.6 ± 57.9	407.7 ± 18.7	416.4 ± 39.5
SpO2 (%)	100.0 ± 0.8	100.0 ± 1.1	100.0 ± 0.5	99.6 ± 1.4	99.7 ± 1.8	99.2 ± 2.1
Temp. (°C)	37.1 ± 0.2	37.1 ± 0.3	36.8 ± 0.2	37.1 ± 0.2	37.0 ± 0.3	36.8 ± 0.2
paO_2_ (mmHg)	191.0 ± 4.6	213.2 ± 23.3	194.2 ± 37.3	163.3 ± 23.44	195.2 ± 22.91	180.6 ± 20.86
paCO_2_ (mmHg)	52.7 ± 2.1	64.5 ± 11.8	62.8 ± 4.8	52.3 ± 4.6	49.5 ± 6.6	63.0 ± 17.6
pH	7.35 ± 0.03	7.23 ± 0.05	7.29 ± 0.02	7.41 ± 0.03	7.40 ± 0.02	7.35 ± 0.10

*BP, mean arterial blood pressure; RR, respiratory rate; HR, heart rate; SPO2, oxygen saturation; Temp, temperature; paO_2_, arterial partial pressure of oxygen; paCO_2_, arterial partial pressure of carbon dioxide. Presented for illustrative purposes only*.

### Gradual Cooling to 33°C Prevents ICP Elevation 24 h Post-Stroke

**Figure 3:**
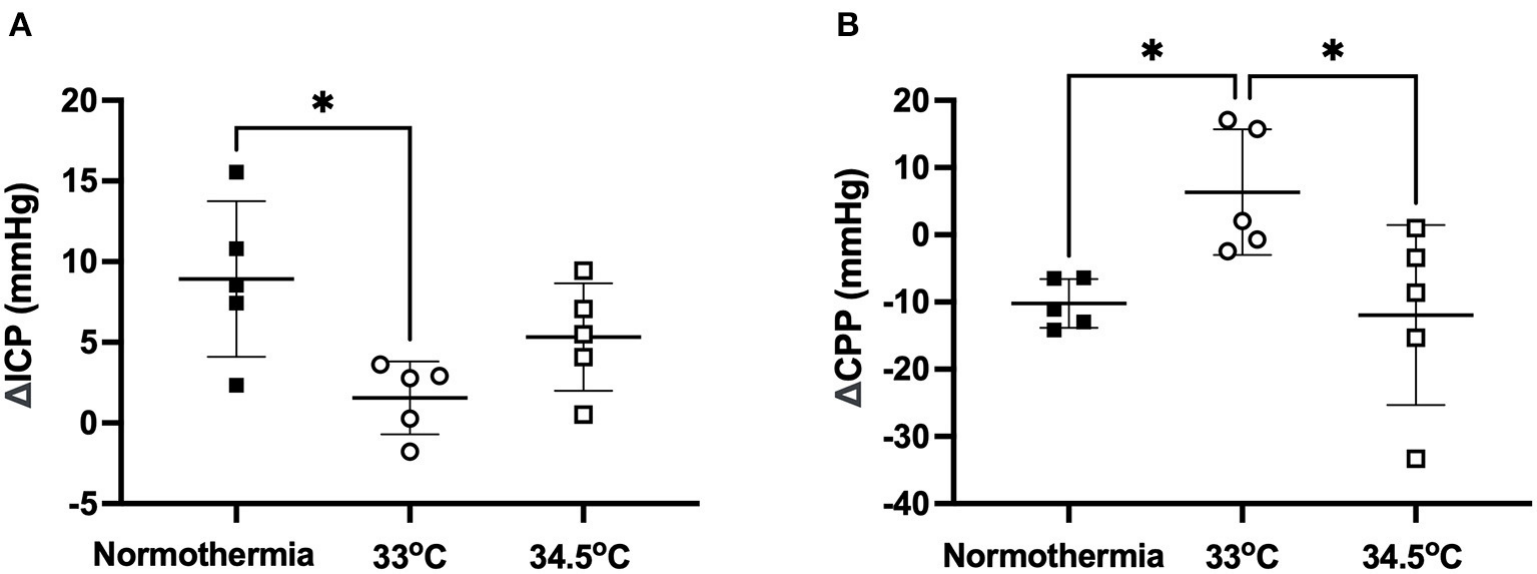
**(A)** Change in ICP from baseline to 24h in the 33°C and 34.5°C hypothermia groups and normothermia control. **(B)** Change in CPP from baseline to 24 h in the 33°C and 34.5°C hypothermia groups and normothermia control. * denotes statistical significance where *p* < 0.05.

ICP rose significantly from baseline to 24 h in the normothermia group (ΔICP = 8.93 ± 4.82 mmHg: Figure 3A). There was a significant main effect between treatment groups, F (2, 12) = 5.1, *p* = 0.02. Tukey *post-hoc* test showed that there was significantly less ICP elevation in the 33°C group compared to normothermia, (ΔICP = 1.56 ± 2.26 mmHg; *p* = 0.02). ICP increased slightly in the 34.5°C group which resulted in a non-significant difference when compared to normothermia (ΔICP = 5.32 ± 3.34 mmHg; *p* = 0.29). CPP decreased significantly from baseline to 24 h in the normothermia group (ΔCPP = −10.21 ± 3.62 mmHg: Figure 3B). There was a significant main effect between treatment groups, F (2, 12) = 5.461, *p* = 0.02. Hypothermia animals in the 33°C group showed an increase in CPP from baseline to 24 h when compared to normothermia (ΔCPP = 6.35 ± 9.33 mmHg, *p* = 0.04). Hypothermia animals in the 34.5°C group showed no difference in CPP when compared with normothermia (ΔCPP = −11.93 ± 13.41mmHg, *p* = 0.96).

### Gradual Cooling to 33°C Reduced Infarct Volume 24 h Post-Stroke

**Figure 4:**
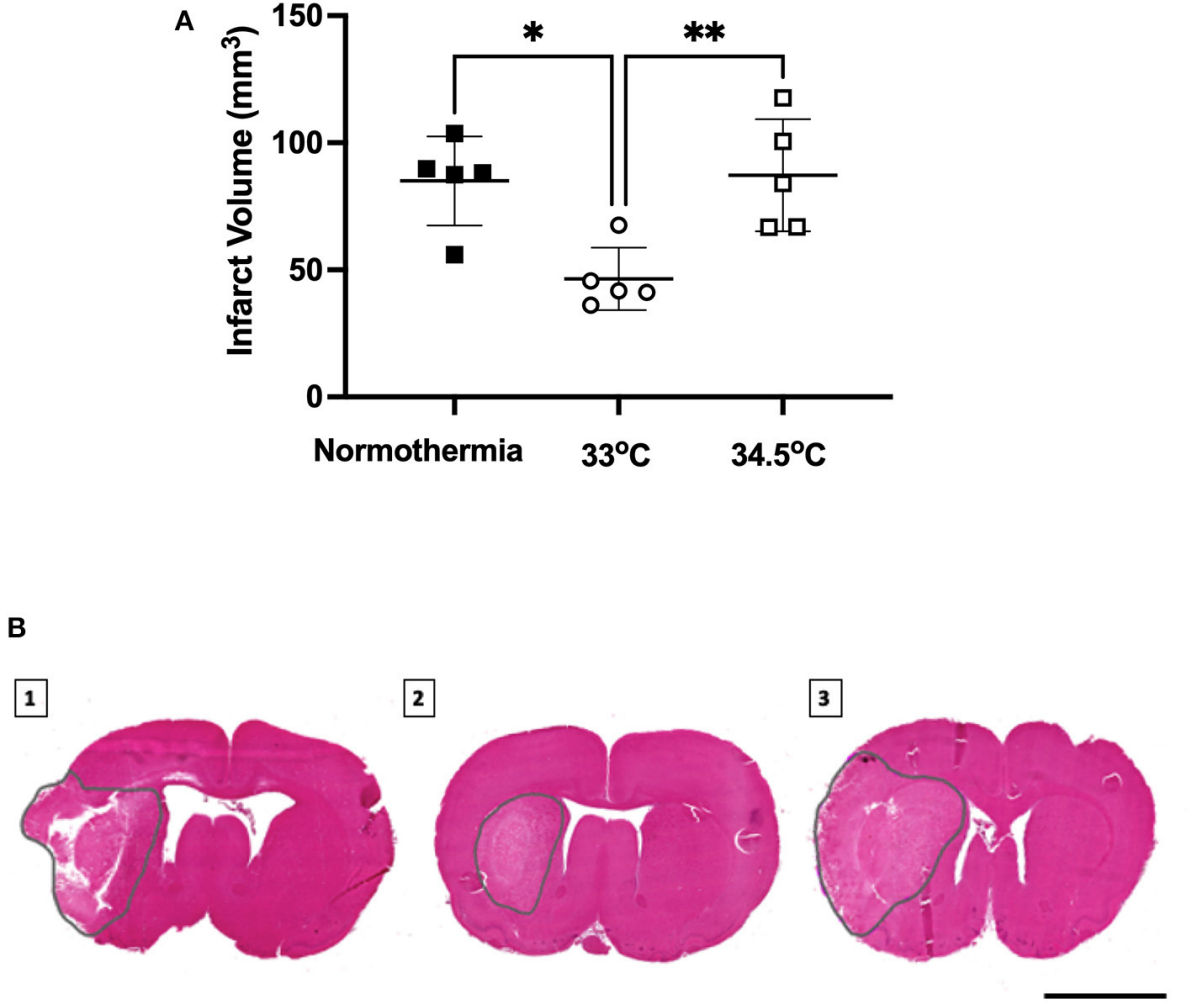
**(A)** Infarct volume from H&E staining at 24 h measured in all groups. * denotes statistical significance where *p* < 0.05, ** denotes statistical significance where *p* < 0.01. **(B)** Representative H&E images of infarct size of (1) normothermia controls, (2) hypothermia 33°C and (3) hypothermia 34.5°C animals at 24 h post-stroke. Dotted gray lines indicate region of infarct. Scale bar = 4 mm.

There was a significant main effect between treatment groups, F (2, 12) = 8.338, *p* = 0.005- (Figure 4). Hypothermia treated animals to 33°C had significantly smaller infarct volumes than normothermia controls (46.4 ± 12.3 mm^3^ and 85.0 ± 17.5 mm^3^, respectively, *p* = 0.01). Hypothermia treated animals to 34.5°C did not have smaller infarct volumes when compared to normothermia controls (87.2 ± 22.1 mm^3^ *p* = 0.98). Representative images are also presented in Figure 4.

## Discussion

In this study, we have shown that clinically achievable gradual cooling to 33°C, with only 30 min at target temperature, prevented significant elevation of ICP and reduced infarct volume 24 h post-stroke. We have previously shown that 2.5 h hypothermia to 32.5°C prevented ICP rise at 24 h in both young adult and aged rats, and in different strains (6, 7). In these previous studies, a target temperature of 32.5°C was achieved within 20 min and rats were maintained at target for 130 min (the remainder of the 2.5 h duration) before being rewarmed. This current study suggests that hypothermia has the same ICP rise prevention properties as previously shown (5–7), even when using a clinically achievable cooling rate with a shorter duration at target temperature.

The results of this study support previous work indicating a likely link between ICP rise prevention and neuroprotection, resulting from hypothermia. Following an ischaemic stroke, the penumbra is kept temporarily viable by perfusion from the collateral vessels. The blood flow to the ischaemic penumbra is known to be dependent on CPP: the difference between mean arterial pressure and ICP. ICP elevation after stroke causes a reduction in CPP, which thereby reduces blood flow to the penumbra and may be responsible for infarct expansion (6, 9). Preliminary data on human stroke patients also demonstrate an ICP rise 24 h post-stroke (8). At the same time, we have shown that a short period of moderate hypothermia in rats prevents ICP rise, even in the presence of large strokes (7). Previous studies have also shown that hypothermia-treated animals with no ICP elevation also tend to have smaller infarct volumes at 24 h than their normothermia counterparts (5, 6).

In this present study, we have shown that hypothermia to 34.5°C did not significantly prevent ICP elevation or reduce infarct volume. These results have important implications because it suggests that a longer duration at target temperature might be necessary for milder hypothermia (≥34.5°C). Previous animal studies have shown that hypothermia to target temperatures as mild as 35°C for 2 h reduces infarct volume (14). However, there does appear to be a depth-response relationship in which lower temperatures provide greater benefit (28, 29). The lack of efficacy seen in the 34.5°C group in this study is likely due to the ultra-short duration for which target temperature was maintained. This finding suggests that, for a much slower cooling rate, either a greater temperature depth or a longer duration at target temperature is likely required to show benefit. However, at a target temperature of 33°C we found that a short duration of 30 min had similar protective effects on ICP elevation and infarct volume to those shown in studies where target temperature is maintained for longer periods (5–7).

While there are some data suggesting that longer durations of cooling may be required for neuroprotection when the onset of hypothermia is delayed (30, 31), it is evident from experimental studies that a prolonged period of treatment is not necessary when hypothermia is initiated shortly after vessel occlusion and during reperfusion. However, additional studies will be required to determine how long the interval from stroke onset to treatment can be, while still maintaining effectiveness. Previous studies by our group (5–7) and this present study have initiated hypothermia 1 h after vessel occlusion and have demonstrated ICP rise prevention at 24 h. The fact that we were still able to see such robust effects with a very short duration at target temperature suggests that perhaps the most important parameter is reaching target temperature and not necessarily the amount of time spent at target. While a 30 min duration at 33°C was used in this study, it is possible that we may have achieved the same results had we cooled to target temperature and immediately rewarmed. This short-duration approach could make hypothermia much more clinically feasible, as the therapy can be initiated at the time of sedation for endovascular thrombectomy, for example.

Our results have demonstrated that clinically relevant cooling rates are feasible and effective in rats. The rate of cooling is an important factor from a translational standpoint and is often not the focus in experimental stroke studies utilizing hypothermia. It has been well documented that humans take considerably longer to cool to target temperature than rodents because of differences in surface area to volume ratio (26). Target temperature is achieved very rapidly in animals; for example, previous work from our group has shown that a target temperature of 32.5°C can be achieved within 20 min (5). This gives us a rate of 0.23°C/min or 13.8°C/h. This is clearly not comparable to human cooling rates, for example the endovascular cooling feasibility study by Georgiadis et al. found that the cooling rate for stroke patients to reach a target temperature of 33°C was 1.4 ± 0.6°C/h (32). The gradual cooling model used in this present study is a major improvement for animal studies with a cooling rate of 2°C/h in the 33°C group and 1°C/h in the 34.5°C group.

We have demonstrated, using a novel method of gradual hypothermia coupled with intensive temperature monitoring, that cooling rates shown to be achieved in humans, can also be achieved in rats. A limitation of this study was that we did not use the same endovascular cooling method that is common in human hypothermia trials. However, there are currently no methods available in small animals that precisely mimic endovascular cooling in humans. Another limitation of this study is that we did not explore whether the robust neuroprotection of hypothermia extends beyond the 24 h observational window. However, the 24 h timepoint is important because it is when ICP elevation peaks post-stroke (5–7). Investigations into the long-term efficacy of hypothermia beyond 24 h will be the focus of future studies. Additionally, further studies will be required to explore the efficacy of short-duration cooling to 34.5°C. This milder temperature may have more robust effects on ICP elevation and infarct volume than was shown in this study if for example, a longer duration at target temperature was used. Neurological deficit assessments were not included in this study. While it is important to determine whether the neuroprotection offered by hypothermia to 33°C extends to neurological deficits, we were powered based on our primary outcome of ΔICP, not neurological deficit scores. Sample size calculations for neurological scores or other behavioral measures would result in much larger group sizes (33).

Lastly, there was a brief occurrence of post-operative hypothermia in all groups in which core body temperature dropped for a short period after rewarming. However, post-operative hypothermia is a universal phenomenon that occurs (34) but is often not measured. While every effort was made to ensure adequate rewarming in the hypothermia groups, the fact that we also saw this dip in temperature occur in our normothermia controls suggest that it is not a factor that influenced the results obtained.

In conclusion, long durations of cooling and their resulting complications have limited large-scale clinical trials and would likely limit the application of long-duration hypothermia as a widely used therapy in stroke. Achieving target temperature has been a challenge in clinical trials of hypothermia in stroke; however, this step may be critical to patient outcome. The lack of benefit seen at 34.5°C highlights the critical importance of achieving target temperature. Recent results from one of the most prominent early-phase clinical trials of hypothermia for ischaemic stroke (EuroHYP-1) found that only 31% of patients recruited achieved target temperature (34–35°C) and suggest this as a potential reason for the lack of benefit reported in the study (35). There is only a 1.5°C difference in target temperature between the two treatments groups in the present study, yet we have shown a dramatic difference in outcome between the groups. This narrow window of effect may also be present in humans, therefore ensuring that target temperature is achieved may have significant impacts on patient outcome. Moreover, the effectiveness of only 30 min at 33°C highlights the fact that much shorter durations of cooling than those used in clinical trials may be effective with early treatment initiation. In recent years, methods have been well established to achieve rapid cooling in patients using whole-body techniques such as endovascular or skin cooling (15). Direct brain cooling strategies such as intranasal (36) and intracarotid cooling (37) are less well established but are under investigation.

Our results suggest that a very short time period at 33°C may be all that is necessary to prevent significant ICP elevation and reduce infarct volume if cooling is initiated early. Further definition of the time window for treatment initiation will be needed, however, these results raise the prospect of benefit from a far more feasible hypothermia paradigm that has previously been tested, and hope that the long-recognized neuroprotective benefits may be able to be realized for stroke patients.

## Data Availability Statement

The raw data supporting the conclusions of this article will be made available by the authors, without undue reservation.

## Ethics Statement

The animal study was reviewed and approved by the Animal Care and Ethics Committee of the University of Newcastle.

## Author Contributions

DO performed the experimental study, analysed and interpreted the data, performed the statistical analyses and drafted the manuscript. NM and SA contributed to the experimental part of the study. DP and SWB were involved in the histological and image analysis for the study. DJB, KC, AP, and NJS conceived the study and participated in its design and coordination. All authors have read and approved the final manuscript.

## Funding

DO was supported by an International Postgraduate Research Scholarship awarded by the University of Newcastle. DJB was supported by the National Health and Medical Research Council Australia (APP1182153). KC was supported by the Hunter Medical Research Institute under the Dalara Early Career Researcher Fellowship. AP was supported by the NSW Ministry of Health under the NSW Health Early-Mid Career Research Fellowship Scheme. NJS was supported by a co-funded Australian National Health and Medical Research Council/National Heart Foundation Career Development/Future Leader Fellowship [GNT1110629/100827].

## Conflict of Interest

The authors declare that the research was conducted in the absence of any commercial or financial relationships that could be construed as a potential conflict of interest.

## Publisher's Note

All claims expressed in this article are solely those of the authors and do not necessarily represent those of their affiliated organizations, or those of the publisher, the editors and the reviewers. Any product that may be evaluated in this article, or claim that may be made by its manufacturer, is not guaranteed or endorsed by the publisher.
